# Thailandepsin A

**DOI:** 10.1107/S1600536811041390

**Published:** 2011-10-12

**Authors:** Cheng Wang, Yi-Qiang Cheng

**Affiliations:** a Department of Biological Sciences, Department of Chemistry and Biochemistry, Univeristy of Wisconsin–Milwaukee, PO Box 413, Milwaukee, WI 53201, USA

## Abstract

Thailandepsin A [systematic name: (*E*)-(1*S*,5*S*,6*R*,9*S*,20*R*)-6-[(2*S*)-butan-2-yl]-5-hy­droxy-20-[2-(meth­yl­sulfan­yl)eth­yl]-2-oxa-11,12-dithia-7,19,22-triaza­bicyclo­[7.7.6]docosa-15-ene-3,8,18,21-tetra­one], C_23_H_37_N_3_O_6_S_3_, is a newly reported [Wang *et al.* (2011). *J. Nat. Prod.* doi:10.1021/np200324x] bicyclic depsipeptide that has potent histone deacetyl­ase inhibitory activity and broad-spectrum anti­proliferative activity. The absolute configuration of thailandepsin A has been determined from the anomalous dispersion and the stereochemistry of all chiral C atoms. Intra­molecular N—H⋯O and N—H⋯S hydrogen bonds occur. Inter­molecular N—H⋯O and O—H⋯O hydrogen bonds are observed in the crystal structure.

## Related literature

For general background to histone deacetyl­ase (HDAC) inhibitors as a new class of anti­cancer agents, see: FDA (2010 ▶); Furumai *et al.* (2002 ▶); Grant *et al.* (2010 ▶); Khan & La Thangue (2008 ▶); Mann *et al.* (2007 ▶); Ueda *et al.* (1994 ▶). For related structures, see: Shigematsu *et al.* (1994 ▶). For geometric data, see: Chou & Blinn (1997 ▶). For the biological activity of the title compound, see: Wang *et al.* (2011 ▶).
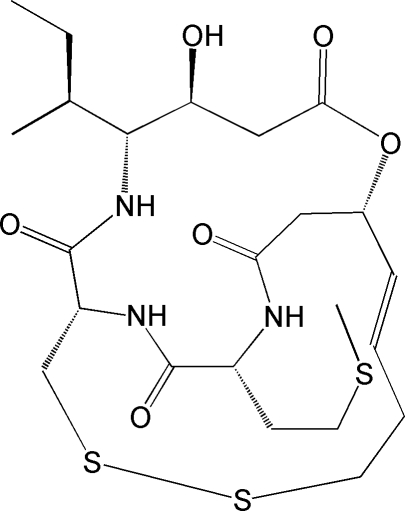


## Experimental

### 

#### Crystal data

C_23_H_37_N_3_O_6_S_3_
*M* *_r_* = 547.74
Orthorhombic, 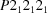

*a* = 12.7747 (3) Å
*b* = 13.2926 (3) Å
*c* = 15.4218 (4) Å
*V* = 2618.76 (11) Å^3^
*Z* = 4
Cu *K*α radiation
μ = 2.96 mm^−1^
*T* = 100 K
0.45 × 0.42 × 0.38 mm

#### Data collection

Bruker SMART APEXII area-detector diffractometer
Absorption correction: multi-scan (*SADABS*; Bruker, 2007 ▶) *T*_min_ = 0.352, *T*_max_ = 0.403
34598 measured reflections
4990 independent reflections
4981 reflections with *I* > 2σ(*I*)
*R*_int_ = 0.025

#### Refinement

*R*[*F* ^2^ > 2σ(*F* ^2^)] = 0.027
*wR*(*F* ^2^) = 0.071
*S* = 1.05
4990 reflections
326 parameters
4 restraints
H atoms treated by a mixture of independent and constrained refinement
Δρ_max_ = 0.35 e Å^−3^
Δρ_min_ = −0.36 e Å^−3^
Absolute structure: Flack (1983 ▶), 2102 Friedel pairs
Flack parameter: 0.000 (9)

### 

Data collection: *APEX2* (Bruker, 2007 ▶); cell refinement: *SAINT* (Bruker, 2007 ▶); data reduction: *SAINT*; program(s) used to solve structure: *SHELXTL* (Sheldrick, 2008 ▶); program(s) used to refine structure: *SHELXTL*; molecular graphics: *SHELXTL* and *OLEX2* (Dolomanov *et al.*, 2009 ▶); software used to prepare material for publication: *SHELXTL*.

## Supplementary Material

Crystal structure: contains datablock(s) I, global. DOI: 10.1107/S1600536811041390/zl2411sup1.cif

Supplementary material file. DOI: 10.1107/S1600536811041390/zl2411Isup2.cdx

Structure factors: contains datablock(s) I. DOI: 10.1107/S1600536811041390/zl2411Isup3.hkl

Supplementary material file. DOI: 10.1107/S1600536811041390/zl2411Isup4.cml

Additional supplementary materials:  crystallographic information; 3D view; checkCIF report

## Figures and Tables

**Table 1:** Hydrogen-bond geometry (Å, °)

*D*—H⋯*A*	*D*—H	H⋯*A*	*D*⋯*A*	*D*—H⋯*A*
O4—H4⋯O5^i^	0.84 (1)	1.90 (1)	2.7394 (15)	176 (2)
N1—H1⋯O6	0.88 (1)	2.06 (1)	2.9203 (17)	166 (2)
N2—H2⋯S2	0.88 (1)	2.83 (2)	3.2491 (13)	111 (2)
N3—H3⋯O4^ii^	0.88 (1)	2.33 (1)	3.1534 (16)	155 (2)

Symmetry codes: (i) 
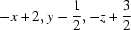
; (ii) 
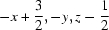
.

**Fig. 1.**
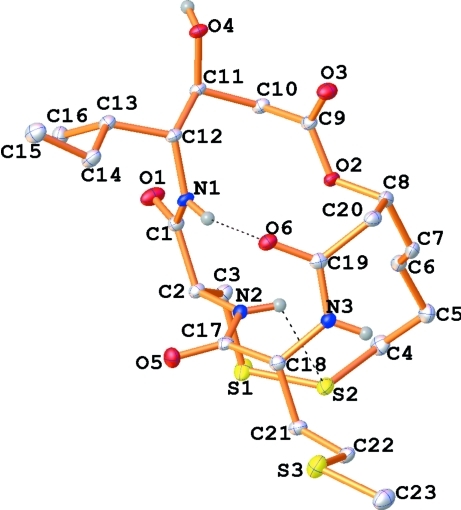
The molecular structure of thailandepsin A with displacement ellipsoids shown at the 50% probability level. For clarity, all H atoms attached to carbon atoms are omitted. Intramolecular hydrogen bonds are shown as dashed lines.

**Fig. 2.**
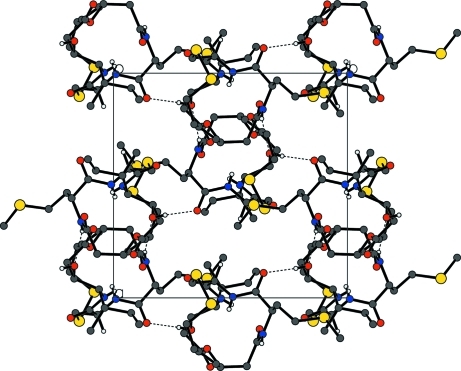
A packing diagram of thailandepsin A, viewed along the c axis. For clarity, all H atoms attached to carbon atoms are omitted. The dashed lines represent hydrogen bonds.

## supplementary crystallographic information

### Comment

With the FDA approval of both SAHA (Vorinostat) and FK228 (Romidepsin) for the treatment of cutaneous T-cell lymphoma (FDA, 2010; Mann *et al.*, 2007), histone deacetylase (HDAC) inhibitors have been in the spotlight in recent years as a new class of anticancer agents (Grant *et al.*, 2010; Khan & La Thangue, 2008). FK228, a natural product produced by *Chromobacterium violaceum* No. 968 (Ueda *et al.*, 1994), represents a family of natural products that contain a signature disulfide bond that is known or presumed to mediate a novel mode of anticancer action in which a reduced thiol group "warhead" chelates a Zn^2+^ in the catalytic center of Class I and Class II HDACs thereby inhibiting the enzyme activities (Furumai *et al.*, 2002; Wang *et al.*, 2011). The crystal structure of FK228 was reported in 1994 (Shigematsu *et al.*, 1994).

Thailandepsin A is a natural analogue of FK228 newly discovered from *Burkholderia thailandensis* E264 by a genomics-guided approach; it has potent histone deacetylase inhibitory activities and broad-spectrum antiproliferative activities (Wang *et al.*, 2011). The chemical structure of thailandepsin A was established by a combination of spectroscopic analyses, chemical derivatization and degradation. Here we report the crystal structure of thailandepsin A.

Thailandepsin A is a bicyclic depsipeptide and consists of four building blocks, *D*-cysteine (*D*-Cys), *D*-methionine (*D*-Met), 4-amino-3-hydroxy-5-methylheptanoic acid (Ahhp, derived from an isoleucine and an acetate unit) and 3-hydroxy-7-mercapto-4-heptenoic acid (Acyl, derived from a cysteine and two acetate units). The primary structure of thailandepsin A is *D*-Met-*D*-Cys-Ahhp-Acyl. X-ray crystallographic analysis indicates that the skeleton of thailandepsin A consists of a [7,7,6] 22-membered ring adopting an uncommon cage-shape that includes a 15-membered macrocyclic lactone and a 15-membered ring and a signature disulfide bond. The bridge ring is almost perpendicular to the main ring and the dihedral angle of these two least-squares planes is 77.7 (1)°. The side chains of methionine and isoleucine have less strain and can freely rotate on the single bonds. In order to obtain minimum energy positions, the alkyl groups arrange so on the molecular skeleton that they point away from each other.

The absolute configurations at C2, C8, C11 and C13 are *S* and the absolute configurations at C12 and 18 are *R* as established based on the results of anomalous dispersion. The geometric isomerism of the double bond in the Acyl component is determined as *E*. The backbone moiety from the carboxyl group of Acyl through methionine and cysteine to the amine group of Ahhp, (Acyl)-CO^1^—Met^2^—Cys^3^—NH^4^-(Ahhp), forms a peculiar secondary structure, a type I' β-turn, and the value of *Ψ* and *Φ* are 57.26 (17)°, 29.76 (18)°, 95.49 (16)° and -18.11 (19)° (Chou & Blinn, 1997). There are two intramolecular and two intermolecular hydrogen bonds present (Table 1, Fig. 1 and 2).

### Experimental

Thailandepsin A was purified from the fermentation broth of *B. thailandensis* E264 as described earlier (Wang *et al.*, 2011). Pure thailandepsin A was dissolved in methanol and block-like crystals were obtained after evaporation of the solvent at room temperature.

### Refinement

All hydrogen atoms attached to the carbon atoms were placed in geometrically idealized positions (C—H = 0.98, 0.99 and 1.00 Å on the primary, secondary and tertiary aliphatic C atoms respectively, 0.95 Å on aromatic C). The H atoms were refined as riding, with isotropic displacement coefficients of *U*_iso_(H) = 1.5*U*_eq_(C) for methyl groups or 1.2*U*_eq_(C) otherwise. The hydrogen atoms attached to N and O were located in difference maps and refined independently with restraints and constraints. The H atoms on N atoms were restrained to have N—H distances of 0.880 (1) Å and their *U*_iso_ values were constrained as equal to 1.2 times the *U*_eq_ of their carrier atoms. The H atom on O was restrained to have an O—H distance of 0.840 (1) Å and the *U*_iso_ value was assigned as equal to 1.5 times the *U*_eq_ of the oxygen atom.

### Crystal data

**Table:**

C_23_H_37_N_3_O_6_S_3_	*F*(000) = 1168
*M**_r_* = 547.74	*D*_x_ = 1.389 Mg m^−^^3^
Orthorhombic, *P*2_1_2_1_2_1_	Cu *K*α radiation, λ = 1.54178 Å
Hall symbol: P 2ac 2ab	Cell parameters from 9793 reflections
*a* = 12.7747 (3) Å	θ = 3.5–71.2°
*b* = 13.2926 (3) Å	µ = 2.96 mm^−^^1^
*c* = 15.4218 (4) Å	*T* = 100 K
*V* = 2618.76 (11) Å^3^	Block, colourless
*Z* = 4	0.45 × 0.42 × 0.38 mm

### Data collection

**Table:**

Bruker SMART APEXII area-detector diffractometer	4990 independent reflections
Radiation source: fine-focus sealed tube	4981 reflections with *I* > 2σ(*I*)
graphite	*R*_int_ = 0.025
0.50° ω and 0.5 ° φ scans	θ_max_ = 71.7°, θ_min_ = 4.4°
Absorption correction: multi-scan (*SADABS*; Bruker, 2007)	*h* = −15→14
*T*_min_ = 0.352, *T*_max_ = 0.403	*k* = −15→16
34598 measured reflections	*l* = −18→17

### Refinement

**Table:**

Refinement on *F*^2^	Secondary atom site location: difference Fourier map
Least-squares matrix: full	Hydrogen site location: inferred from neighbouring sites
*R*[*F*^2^ > 2σ(*F*^2^)] = 0.027	H atoms treated by a mixture of independent and constrained refinement
*wR*(*F*^2^) = 0.071	*w* = 1/[σ^2^(*F*_o_^2^) + (0.051*P*)^2^ + 0.7593*P*] where *P* = (*F*_o_^2^ + 2*F*_c_^2^)/3
*S* = 1.05	(Δ/σ)_max_ = 0.001
4990 reflections	Δρ_max_ = 0.35 e Å^−^^3^
326 parameters	Δρ_min_ = −0.36 e Å^−^^3^
4 restraints	Absolute structure: Flack (1983), ???? Friedel pairs
Primary atom site location: structure-invariant direct methods	Flack parameter: 0.000 (9)

### Special details

**Table:**

Geometry. All e.s.d.'s (except the e.s.d. in the dihedral angle between two l.s. planes) are estimated using the full covariance matrix. The cell e.s.d.'s are taken into account individually in the estimation of e.s.d.'s in distances, angles and torsion angles; correlations between e.s.d.'s in cell parameters are only used when they are defined by crystal symmetry. An approximate (isotropic) treatment of cell e.s.d.'s is used for estimating e.s.d.'s involving l.s. planes.
Refinement. Refinement of *F*^2^ against ALL reflections. The weighted *R*-factor *wR* and goodness of fit *S* are based on *F*^2^, conventional *R*-factors *R* are based on *F*, with *F* set to zero for negative *F*^2^. The threshold expression of *F*^2^ > σ(*F*^2^) is used only for calculating *R*-factors(gt) *etc*. and is not relevant to the choice of reflections for refinement. *R*-factors based on *F*^2^ are statistically about twice as large as those based on *F*, and *R*- factors based on ALL data will be even larger.

### Fractional atomic coordinates and isotropic or equivalent isotropic displacement parameters (Å^2^)

**Table:**

	*x*	*y*	*z*	*U*_iso_*/*U*_eq_	
S1	1.10290 (3)	−0.14637 (3)	0.38774 (2)	0.02184 (10)	
S2	0.97356 (3)	−0.08216 (3)	0.33328 (2)	0.01709 (9)	
S3	0.91337 (3)	0.40012 (3)	0.40152 (3)	0.02362 (10)	
O1	1.08885 (10)	−0.18335 (9)	0.67591 (8)	0.0223 (3)	
O2	0.77376 (8)	−0.09984 (8)	0.61157 (7)	0.0141 (2)	
O3	0.69383 (9)	−0.08629 (9)	0.74184 (7)	0.0190 (2)	
O4	0.85790 (9)	−0.16775 (8)	0.88174 (7)	0.0140 (2)	
H4	0.8699 (17)	−0.2254 (7)	0.9016 (13)	0.021*	
O5	1.11125 (9)	0.14154 (8)	0.55597 (7)	0.0179 (2)	
O6	0.86120 (9)	0.12479 (8)	0.64747 (7)	0.0158 (2)	
N1	0.99720 (10)	−0.04020 (10)	0.70173 (8)	0.0128 (3)	
H1	0.9652 (14)	0.0126 (9)	0.6798 (12)	0.015*	
N2	1.00458 (10)	0.00984 (9)	0.52676 (8)	0.0121 (2)	
H2	0.9444 (8)	−0.0082 (14)	0.5038 (12)	0.014*	
N3	0.83857 (10)	0.13784 (10)	0.50282 (8)	0.0138 (3)	
H3	0.7955 (12)	0.1335 (15)	0.4584 (8)	0.017*	
C1	1.05386 (12)	−0.10227 (12)	0.65205 (10)	0.0143 (3)	
C2	1.07489 (12)	−0.06840 (11)	0.55727 (9)	0.0131 (3)	
H2A	1.1482	−0.0423	0.5537	0.016*	
C3	1.06714 (13)	−0.16258 (12)	0.50086 (10)	0.0174 (3)	
H3A	0.9943	−0.1877	0.5034	0.021*	
H3B	1.1128	−0.2151	0.5262	0.021*	
C4	0.88346 (14)	−0.18723 (13)	0.31638 (11)	0.0202 (3)	
H4A	0.8850	−0.2082	0.2548	0.024*	
H4B	0.9052	−0.2453	0.3524	0.024*	
C5	0.77251 (12)	−0.15466 (13)	0.34132 (11)	0.0190 (3)	
H5A	0.7542	−0.0927	0.3091	0.023*	
H5B	0.7225	−0.2079	0.3240	0.023*	
C6	0.76184 (12)	−0.13526 (13)	0.43705 (10)	0.0173 (3)	
H6	0.7980	−0.1793	0.4752	0.021*	
C7	0.70632 (12)	−0.06202 (12)	0.47258 (10)	0.0162 (3)	
H7	0.6720	−0.0170	0.4341	0.019*	
C8	0.69224 (12)	−0.04303 (12)	0.56827 (10)	0.0154 (3)	
H8	0.6227	−0.0704	0.5865	0.018*	
C9	0.76158 (12)	−0.12126 (11)	0.69723 (10)	0.0136 (3)	
C10	0.84280 (12)	−0.19799 (11)	0.72498 (10)	0.0138 (3)	
H10A	0.8877	−0.2133	0.6743	0.017*	
H10B	0.8059	−0.2608	0.7409	0.017*	
C11	0.91409 (12)	−0.16801 (11)	0.80094 (9)	0.0127 (3)	
H11	0.9724	−0.2180	0.8051	0.015*	
C12	0.96190 (12)	−0.06200 (11)	0.79096 (9)	0.0125 (3)	
H12	0.9029	−0.0143	0.8019	0.015*	
C13	1.04416 (12)	−0.03790 (11)	0.86101 (10)	0.0146 (3)	
H13	1.0124	−0.0559	0.9182	0.017*	
C14	1.06664 (13)	0.07539 (12)	0.86315 (10)	0.0184 (3)	
H14A	1.1138	0.0927	0.8144	0.022*	
H14B	1.0002	0.1125	0.8548	0.022*	
C15	1.11702 (14)	0.10927 (13)	0.94810 (11)	0.0222 (3)	
H15A	1.0677	0.0988	0.9960	0.033*	
H15B	1.1350	0.1808	0.9443	0.033*	
H15C	1.1807	0.0700	0.9585	0.033*	
C16	1.14602 (12)	−0.09806 (13)	0.85283 (10)	0.0184 (3)	
H16A	1.1848	−0.0752	0.8017	0.028*	
H16B	1.1296	−0.1697	0.8467	0.028*	
H16C	1.1888	−0.0878	0.9048	0.028*	
C17	1.02739 (12)	0.10824 (11)	0.52881 (9)	0.0134 (3)	
C18	0.94399 (12)	0.17890 (11)	0.49209 (10)	0.0138 (3)	
H18	0.9480	0.2442	0.5241	0.017*	
C19	0.80478 (12)	0.11430 (11)	0.58346 (10)	0.0141 (3)	
C20	0.69604 (12)	0.06994 (12)	0.59060 (10)	0.0160 (3)	
H20A	0.6486	0.1068	0.5510	0.019*	
H20B	0.6701	0.0797	0.6505	0.019*	
C21	0.96752 (13)	0.19916 (11)	0.39575 (10)	0.0158 (3)	
H21A	1.0409	0.2224	0.3904	0.019*	
H21B	0.9613	0.1351	0.3634	0.019*	
C22	0.89580 (13)	0.27705 (12)	0.35353 (10)	0.0168 (3)	
H22A	0.8220	0.2558	0.3605	0.020*	
H22B	0.9112	0.2807	0.2907	0.020*	
C23	0.82077 (15)	0.46899 (14)	0.33643 (12)	0.0250 (4)	
H23A	0.8190	0.5393	0.3554	0.037*	
H23B	0.7510	0.4392	0.3431	0.037*	
H23C	0.8419	0.4659	0.2754	0.037*	

### Atomic displacement parameters (Å^2^)

**Table:**

	*U*^11^	*U*^22^	*U*^33^	*U*^12^	*U*^13^	*U*^23^
S1	0.01829 (19)	0.0340 (2)	0.01321 (18)	0.00771 (17)	−0.00022 (14)	−0.00696 (15)
S2	0.01714 (18)	0.02111 (19)	0.01302 (16)	0.00009 (15)	−0.00063 (14)	−0.00078 (14)
S3	0.0279 (2)	0.0207 (2)	0.0222 (2)	0.00109 (16)	−0.00648 (16)	0.00032 (16)
O1	0.0286 (6)	0.0210 (6)	0.0172 (6)	0.0118 (5)	0.0048 (5)	0.0045 (5)
O2	0.0140 (5)	0.0171 (5)	0.0112 (5)	0.0017 (4)	0.0001 (4)	0.0034 (4)
O3	0.0199 (5)	0.0216 (6)	0.0156 (5)	0.0040 (5)	0.0037 (4)	0.0017 (5)
O4	0.0190 (5)	0.0131 (5)	0.0098 (5)	0.0019 (4)	0.0019 (4)	0.0018 (4)
O5	0.0167 (5)	0.0166 (5)	0.0205 (5)	−0.0020 (4)	−0.0032 (5)	−0.0018 (5)
O6	0.0197 (5)	0.0159 (5)	0.0119 (5)	0.0022 (4)	0.0004 (4)	−0.0001 (4)
N1	0.0161 (6)	0.0129 (6)	0.0093 (6)	0.0026 (5)	−0.0002 (5)	0.0029 (5)
N2	0.0122 (6)	0.0137 (6)	0.0103 (6)	−0.0004 (5)	−0.0003 (4)	−0.0003 (5)
N3	0.0139 (6)	0.0155 (6)	0.0121 (6)	0.0005 (5)	−0.0008 (5)	0.0010 (5)
C1	0.0145 (7)	0.0156 (7)	0.0129 (7)	0.0005 (6)	0.0002 (5)	0.0013 (6)
C2	0.0146 (7)	0.0142 (7)	0.0104 (6)	0.0017 (6)	0.0001 (5)	−0.0004 (6)
C3	0.0227 (7)	0.0153 (7)	0.0142 (7)	0.0037 (6)	−0.0015 (6)	−0.0014 (6)
C4	0.0243 (8)	0.0191 (7)	0.0170 (8)	−0.0014 (7)	−0.0020 (7)	−0.0043 (6)
C5	0.0184 (8)	0.0213 (8)	0.0172 (8)	−0.0030 (6)	−0.0031 (6)	−0.0015 (6)
C6	0.0167 (7)	0.0196 (7)	0.0156 (7)	−0.0045 (6)	−0.0035 (6)	0.0037 (6)
C7	0.0150 (7)	0.0194 (7)	0.0143 (7)	−0.0019 (6)	−0.0042 (6)	0.0040 (6)
C8	0.0126 (7)	0.0182 (7)	0.0154 (7)	0.0001 (6)	−0.0011 (6)	0.0038 (6)
C9	0.0165 (7)	0.0135 (7)	0.0107 (6)	−0.0032 (6)	−0.0007 (6)	0.0002 (5)
C10	0.0183 (7)	0.0118 (7)	0.0111 (7)	0.0006 (6)	−0.0002 (6)	−0.0001 (5)
C11	0.0157 (7)	0.0136 (7)	0.0087 (6)	0.0015 (6)	0.0001 (6)	0.0010 (5)
C12	0.0152 (7)	0.0133 (6)	0.0089 (6)	0.0014 (6)	0.0010 (6)	0.0009 (5)
C13	0.0176 (7)	0.0156 (7)	0.0106 (7)	0.0009 (6)	−0.0008 (6)	0.0000 (5)
C14	0.0249 (8)	0.0155 (7)	0.0148 (7)	−0.0014 (6)	−0.0034 (6)	0.0017 (6)
C15	0.0287 (9)	0.0189 (8)	0.0190 (8)	−0.0025 (7)	−0.0021 (7)	−0.0013 (6)
C16	0.0161 (7)	0.0208 (8)	0.0182 (8)	0.0011 (6)	−0.0020 (6)	0.0011 (6)
C17	0.0164 (7)	0.0158 (7)	0.0079 (6)	−0.0003 (6)	0.0022 (6)	−0.0004 (5)
C18	0.0153 (7)	0.0133 (7)	0.0127 (7)	−0.0014 (6)	0.0000 (6)	0.0000 (6)
C19	0.0160 (7)	0.0119 (7)	0.0144 (7)	0.0037 (6)	0.0017 (6)	0.0005 (6)
C20	0.0154 (7)	0.0184 (7)	0.0142 (7)	0.0036 (6)	0.0020 (6)	0.0026 (6)
C21	0.0192 (7)	0.0154 (7)	0.0128 (7)	−0.0013 (6)	0.0012 (6)	0.0013 (6)
C22	0.0210 (8)	0.0171 (7)	0.0122 (7)	−0.0029 (6)	−0.0026 (6)	0.0015 (5)
C23	0.0259 (8)	0.0281 (9)	0.0209 (8)	0.0081 (7)	0.0006 (7)	0.0028 (7)

### Geometric parameters (Å, °)

**Table:**

S1—C3	1.8162 (17)	C8—C20	1.541 (2)
S1—S2	2.0406 (6)	C8—H8	1.0000
S2—C4	1.8285 (17)	C9—C10	1.517 (2)
S3—C23	1.8014 (18)	C10—C11	1.536 (2)
S3—C22	1.8095 (16)	C10—H10A	0.9900
O1—C1	1.223 (2)	C10—H10B	0.9900
O2—C9	1.3602 (18)	C11—C12	1.543 (2)
O2—C8	1.4494 (18)	C11—H11	1.0000
O3—C9	1.199 (2)	C12—C13	1.541 (2)
O4—C11	1.4380 (17)	C12—H12	1.0000
O4—H4	0.8399 (10)	C13—C16	1.533 (2)
O5—C17	1.233 (2)	C13—C14	1.533 (2)
O6—C19	1.230 (2)	C13—H13	1.0000
N1—C1	1.338 (2)	C14—C15	1.528 (2)
N1—C12	1.4769 (18)	C14—H14A	0.9900
N1—H1	0.8797 (10)	C14—H14B	0.9900
N2—C17	1.340 (2)	C15—H15A	0.9800
N2—C2	1.4524 (19)	C15—H15B	0.9800
N2—H2	0.8798 (10)	C15—H15C	0.9800
N3—C19	1.353 (2)	C16—H16A	0.9800
N3—C18	1.4624 (18)	C16—H16B	0.9800
N3—H3	0.8799 (10)	C16—H16C	0.9800
C1—C2	1.553 (2)	C17—C18	1.529 (2)
C2—C3	1.528 (2)	C18—C21	1.540 (2)
C2—H2A	1.0000	C18—H18	1.0000
C3—H3A	0.9900	C19—C20	1.513 (2)
C3—H3B	0.9900	C20—H20A	0.9900
C4—C5	1.531 (2)	C20—H20B	0.9900
C4—H4A	0.9900	C21—C22	1.528 (2)
C4—H4B	0.9900	C21—H21A	0.9900
C5—C6	1.505 (2)	C21—H21B	0.9900
C5—H5A	0.9900	C22—H22A	0.9900
C5—H5B	0.9900	C22—H22B	0.9900
C6—C7	1.323 (2)	C23—H23A	0.9800
C6—H6	0.9500	C23—H23B	0.9800
C7—C8	1.508 (2)	C23—H23C	0.9800
C7—H7	0.9500		
			
C3—S1—S2	103.96 (6)	C12—C11—H11	108.5
C4—S2—S1	104.41 (6)	N1—C12—C13	113.84 (12)
C23—S3—C22	98.63 (8)	N1—C12—C11	113.14 (12)
C9—O2—C8	118.31 (12)	C13—C12—C11	112.95 (12)
C11—O4—H4	102.9 (15)	N1—C12—H12	105.3
C1—N1—C12	125.27 (13)	C13—C12—H12	105.3
C1—N1—H1	121.5 (13)	C11—C12—H12	105.3
C12—N1—H1	111.9 (13)	C16—C13—C14	110.81 (13)
C17—N2—C2	123.82 (13)	C16—C13—C12	114.39 (13)
C17—N2—H2	117.7 (13)	C14—C13—C12	110.31 (12)
C2—N2—H2	118.4 (13)	C16—C13—H13	107.0
C19—N3—C18	118.95 (13)	C14—C13—H13	107.0
C19—N3—H3	120.1 (13)	C12—C13—H13	107.0
C18—N3—H3	120.8 (13)	C15—C14—C13	112.76 (13)
O1—C1—N1	124.65 (14)	C15—C14—H14A	109.0
O1—C1—C2	118.38 (14)	C13—C14—H14A	109.0
N1—C1—C2	116.97 (13)	C15—C14—H14B	109.0
N2—C2—C3	111.24 (12)	C13—C14—H14B	109.0
N2—C2—C1	113.92 (12)	H14A—C14—H14B	107.8
C3—C2—C1	106.69 (12)	C14—C15—H15A	109.5
N2—C2—H2A	108.3	C14—C15—H15B	109.5
C3—C2—H2A	108.3	H15A—C15—H15B	109.5
C1—C2—H2A	108.3	C14—C15—H15C	109.5
C2—C3—S1	115.69 (11)	H15A—C15—H15C	109.5
C2—C3—H3A	108.4	H15B—C15—H15C	109.5
S1—C3—H3A	108.4	C13—C16—H16A	109.5
C2—C3—H3B	108.4	C13—C16—H16B	109.5
S1—C3—H3B	108.4	H16A—C16—H16B	109.5
H3A—C3—H3B	107.4	C13—C16—H16C	109.5
C5—C4—S2	109.33 (11)	H16A—C16—H16C	109.5
C5—C4—H4A	109.8	H16B—C16—H16C	109.5
S2—C4—H4A	109.8	O5—C17—N2	123.19 (14)
C5—C4—H4B	109.8	O5—C17—C18	120.72 (13)
S2—C4—H4B	109.8	N2—C17—C18	116.05 (13)
H4A—C4—H4B	108.3	N3—C18—C17	111.75 (12)
C6—C5—C4	112.26 (13)	N3—C18—C21	110.75 (12)
C6—C5—H5A	109.2	C17—C18—C21	109.20 (12)
C4—C5—H5A	109.2	N3—C18—H18	108.4
C6—C5—H5B	109.2	C17—C18—H18	108.4
C4—C5—H5B	109.2	C21—C18—H18	108.4
H5A—C5—H5B	107.9	O6—C19—N3	121.62 (14)
C7—C6—C5	125.51 (15)	O6—C19—C20	121.58 (14)
C7—C6—H6	117.2	N3—C19—C20	116.73 (14)
C5—C6—H6	117.2	C19—C20—C8	113.10 (13)
C6—C7—C8	126.32 (14)	C19—C20—H20A	109.0
C6—C7—H7	116.8	C8—C20—H20A	109.0
C8—C7—H7	116.8	C19—C20—H20B	109.0
O2—C8—C7	106.14 (12)	C8—C20—H20B	109.0
O2—C8—C20	112.45 (12)	H20A—C20—H20B	107.8
C7—C8—C20	112.21 (13)	C22—C21—C18	114.37 (13)
O2—C8—H8	108.6	C22—C21—H21A	108.7
C7—C8—H8	108.6	C18—C21—H21A	108.7
C20—C8—H8	108.6	C22—C21—H21B	108.7
O3—C9—O2	123.95 (14)	C18—C21—H21B	108.7
O3—C9—C10	126.34 (14)	H21A—C21—H21B	107.6
O2—C9—C10	109.66 (12)	C21—C22—S3	111.34 (11)
C9—C10—C11	116.50 (12)	C21—C22—H22A	109.4
C9—C10—H10A	108.2	S3—C22—H22A	109.4
C11—C10—H10A	108.2	C21—C22—H22B	109.4
C9—C10—H10B	108.2	S3—C22—H22B	109.4
C11—C10—H10B	108.2	H22A—C22—H22B	108.0
H10A—C10—H10B	107.3	S3—C23—H23A	109.5
O4—C11—C10	111.44 (12)	S3—C23—H23B	109.5
O4—C11—C12	106.37 (11)	H23A—C23—H23B	109.5
C10—C11—C12	113.29 (12)	S3—C23—H23C	109.5
O4—C11—H11	108.5	H23A—C23—H23C	109.5
C10—C11—H11	108.5	H23B—C23—H23C	109.5
			
C3—S1—S2—C4	−79.45 (8)	O4—C11—C12—N1	−164.12 (12)
C12—N1—C1—O1	−4.2 (2)	C10—C11—C12—N1	−41.37 (17)
C12—N1—C1—C2	175.04 (13)	O4—C11—C12—C13	64.71 (15)
C17—N2—C2—C3	−143.88 (14)	C10—C11—C12—C13	−172.53 (12)
C17—N2—C2—C1	95.49 (16)	N1—C12—C13—C16	−61.27 (17)
O1—C1—C2—N2	161.20 (14)	C11—C12—C13—C16	69.54 (16)
N1—C1—C2—N2	−18.11 (19)	N1—C12—C13—C14	64.44 (16)
O1—C1—C2—C3	38.06 (19)	C11—C12—C13—C14	−164.75 (12)
N1—C1—C2—C3	−141.25 (14)	C16—C13—C14—C15	−71.70 (17)
N2—C2—C3—S1	61.75 (15)	C12—C13—C14—C15	160.60 (13)
C1—C2—C3—S1	−173.45 (10)	C2—N2—C17—O5	0.9 (2)
S2—S1—C3—C2	−79.42 (12)	C2—N2—C17—C18	178.86 (12)
S1—S2—C4—C5	138.27 (10)	C19—N3—C18—C17	57.26 (17)
S2—C4—C5—C6	−67.15 (16)	C19—N3—C18—C21	179.24 (13)
C4—C5—C6—C7	141.33 (16)	O5—C17—C18—N3	−152.25 (13)
C5—C6—C7—C8	178.20 (15)	N2—C17—C18—N3	29.76 (18)
C9—O2—C8—C7	−160.50 (12)	O5—C17—C18—C21	84.87 (16)
C9—O2—C8—C20	76.45 (16)	N2—C17—C18—C21	−93.11 (15)
C6—C7—C8—O2	16.4 (2)	C18—N3—C19—O6	−1.4 (2)
C6—C7—C8—C20	139.65 (16)	C18—N3—C19—C20	−178.68 (12)
C8—O2—C9—O3	−9.1 (2)	O6—C19—C20—C8	−97.53 (17)
C8—O2—C9—C10	168.50 (12)	N3—C19—C20—C8	79.73 (16)
O3—C9—C10—C11	−58.7 (2)	O2—C8—C20—C19	47.62 (17)
O2—C9—C10—C11	123.75 (13)	C7—C8—C20—C19	−71.95 (17)
C9—C10—C11—O4	71.69 (16)	N3—C18—C21—C22	62.21 (16)
C9—C10—C11—C12	−48.21 (17)	C17—C18—C21—C22	−174.33 (13)
C1—N1—C12—C13	84.41 (18)	C18—C21—C22—S3	64.71 (15)
C1—N1—C12—C11	−46.31 (19)	C23—S3—C22—C21	179.27 (12)

### Hydrogen-bond geometry (Å, °)

**Table:**

*D*—H···*A*	*D*—H	H···*A*	*D*···*A*	*D*—H···*A*
O4—H4···O5^i^	0.84 (1)	1.90 (1)	2.7394 (15)	176 (2)
N1—H1···O6	0.88 (1)	2.06 (1)	2.9203 (17)	166.(2)
N2—H2···S2	0.88 (1)	2.83 (2)	3.2491 (13)	111.(2)
N3—H3···O4^ii^	0.88 (1)	2.33 (1)	3.1534 (16)	155.(2)

Symmetry codes: (i) −*x*+2, *y*−1/2, −*z*+3/2; (ii) −*x*+3/2, −*y*, *z*−1/2.
